# Low donor chimerism may be sufficient to prevent demyelination in adrenoleukodystrophy

**DOI:** 10.1002/jmd2.12259

**Published:** 2021-11-17

**Authors:** Takahiro Ikeda, Yuta Kawahara, Akihiko Miyauchi, Hitomi Niijima, Rieko Furukawa, Nobuyuki Shimozawa, Akira Morimoto, Hitoshi Osaka, Takanori Yamagata

**Affiliations:** ^1^ Department of Pediatrics Jichi Medical University Tochigi Japan; ^2^ Department of Radiology Jichi Medical University Tochigi Japan; ^3^ Division of Genomics Research, Life Science Research Center Gifu University Gifu Japan

**Keywords:** ATP‐binding cassette subfamily D member 1, cerebral adrenoleukodystrophy, cerebrospinal fluid, hematopoietic stem cell transplantation, unrelated cord blood transplantation, very‐long‐chain fatty acids

## Abstract

Adrenoleukodystrophy (ALD) is a peroxisomal disorder characterized by white matter degeneration caused by adenosine triphosphate‐binding cassette subfamily D member 1 (*ABCD1*) gene mutations, which lead to an accumulation of very‐long‐chain fatty acids (VLCFA). Hematopoietic stem cell transplantation (HSCT) is the most effective treatment; however, the ratio of donor‐to‐recipient cells required to prevent the progression of demyelination is unclear. The proband was diagnosed with the childhood cerebral form of ALD at 5 years of age based on the clinical phenotype, elevated plasma VLCFA levels, and pathogenic *ABCD1* mutation c.293C>T (p.Ser98Leu). Soon after the diagnosis, he became bedridden. At 1 year of age, his younger brother was found to carry the same *ABCD1* mutation; despite being asymptomatic, at 1 year and 9 months, head magnetic resonance imaging (MRI) showed high‐signal‐intensity lesions in the cerebral white matter. The patient underwent unrelated cord blood transplantation (UCBT) with a reduced conditioning regimen, which resulted in mixed chimerism. For 7 years after UCBT, the donor chimerism remained low (<10%) in peripheral blood and cerebrospinal fluid. However, even though a second HSCT was not performed, his neurological symptoms and brain MRI findings did not deteriorate. Our case suggests that even a small number of donor cells may prevent demyelination in ALD. This is an important case when considering the timing of a second HSCT.

**Synopsis**

Unrelated cord blood transplant may prevent neurologic complications of childhood cerebral adrenoleukodystrophy despite low chimerism in peripheral blood and cerebrospinal fluid.

## 1 INTRODUCTION

Adrenoleukodystrophy (ALD) (OMIM #300100) is an X‐linked peroxisomal disease with an incidence estimated to be 1 in 20 000–30 000 live births.^1^ It is caused by mutations in the adenosine triphosphate (ATP)‐binding cassette subfamily D member 1 (*ABCD1*) gene, which leads to the accumulation of very‐long‐chain fatty acids (VLCFAs).^2^ Based on the disease course, ALD is classified into three main types: cerebral adrenoleukodystrophy (CALD), myelopathy, and Addison's disease. CALD has the most severe phenotype, in which VLCFA accumulation induces a localized aggressive inflammatory response that results in extensive demyelination of the cerebral white matter, causing progressive neurological regression. CALD is further divided into subtypes based on the patients' age of onset: childhood, adolescence, and adult‐onset cerebral ALD. Without therapeutic intervention, childhood CALD (CCALD) typically follows a fatal course within the first decade of life.^3^

Although various therapies have been attempted, including Lorenzo's oil as part of a VLCFA‐restricted diet, hematopoietic stem cell transplantation (HSCT) is the most effective treatment for patients with CCALD as it shows long‐term beneficial effects.^4^, ^5^, ^6^, ^7^, ^8^ Furthermore, current research emphasizes that early HSCT following the onset of CCALD, even in the presymptomatic period, can preserve neurological function.^7^, ^8^, ^9^, ^10^

Several studies have recently demonstrated the use of unrelated cord blood as a donor source for HSCT in CCALD.^11^, ^12^, ^13^ Unrelated cord blood transplantation (UCBT) has benefited patients with urgent care needs, permitting a high degree of human leukocyte antigen (HLA) mismatch and thus, a low risk of graft‐vs‐host disease (GVHD).^14^ However, this procedure involves a higher risk of graft failure or mixed chimerism, hence necessitating a second HSCT that is often associated with transplant‐related mortality. The chimeric ratio of donor‐to‐recipient cells required to prevent the progression of central nervous system (CNS) injury and avoid a second HSCT has yet to be determined.

Herein, we report a case of CCALD with donor cells persistently detected in the cerebrospinal fluid (CSF). In this patient, CNS lesions did not progress, even with low donor chimerism both in peripheral blood (PB) and CSF after UCBT.

## 2 CASE REPORT

The patient was an 8‐year‐old boy born to unrelated parents. His mother suffered from mild visual impairment and pigment deficiency. The proband (the patient's elder brother) developed visual impairment and developmental regression at 5 years of age. Brain magnetic resonance imaging (MRI) showed bilateral diffuse high‐intensity areas on T2 weighted images in the posterior deep white matter. Plasma VLCFA levels were elevated, and Sanger sequencing showed that the *ABCD1* gene carried a pathogenic missense mutation, c.293C>T (p.Ser98Leu).^15^ His mother was a carrier of this missense mutation. Based on these results, the patient's elder brother was diagnosed with ALD. Four months after the diagnosis, he became bedridden and needed tube feeding. Therefore, we did not perform HSCT on him.

After the elder brother's diagnosis, the patient was found to have the same mutation in *ABCD1* at 1 year and 4 months of age. His growth and development were normal, and no neurological symptoms were noted. The plasma levels of VLCFAs were elevated: C24:0/C22:0 1.55 (average, 1.05 ± 0.16 SD), C25:0/C22:0 0.084 (average, 0.024 ± 0.006 SD), and C26:0/C22:0 0.075 (average 0.012 ± 0.005 SD). Plasma cortisol was within the normal limits (8.9 μg/dl), but plasma adrenocorticotropic hormone had increased to 271 pg/ml (normal range, 7.2–63.3). He was diagnosed with adrenal deficiency, and oral corticosteroid therapy was started.

**FIGURE 1:**
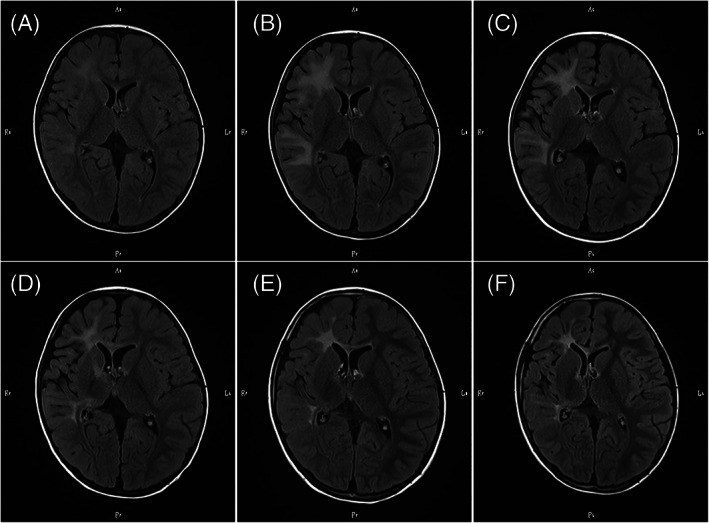
Change in axial fluid‐attenuated inversion recovery images on brain magnetic resonance imaging (MRI). (A) MRI scan at 1 year and 7 months of age, 2 months before unrelated cord blood transplantation (UCBT). Mild high‐signal intensity can be noted in the white matter of the right frontal and temporal areas. (B) MRI scan at 1 year and 9 months of age, 3 days before UCBT. The image shows expanded hyperintense lesions. (C) MRI scan at 1 year and 11 months of age, 2 months after UCBT. The scan revealed mild atrophy of the right frontal and temporal areas. (D) MRI scan at 2 years and 5 months of age, 8 months after UCBT. The image shows a slight decrease in right periventricular white matter. (E) and (F) MRI scan at 4 years of age, 3 years after UCBT and at 8 years of age, 7 years after UCBT. No progression was noted

Although he showed no neurological symptoms, a brain MRI showed mild high‐signal‐intensity lesions in the white matter of the right frontal and temporal areas at 1 year and 7 months. The patient had a Loes score for MRI findings of 3.0 points (Figure 1A).^16^ Two months later, the white matter lesion progressed, and atrophy of the right hemisphere was noted. The Loes score had increased to 4.5 points (Figure 1B), and no contrast effect was observed in either MRI. He underwent UCBT from a sex‐mismatched HLA 8/8 matched donor aged 1 year and 9 months. The conditioning regimen was 3 Gy of total body irradiation, 125 mg/m^2^ fludarabine, and 140 mg/m^2^ melphalan. The GVHD prophylaxis consisted of cyclosporine and short‐term methotrexate.

**FIGURE 2:**
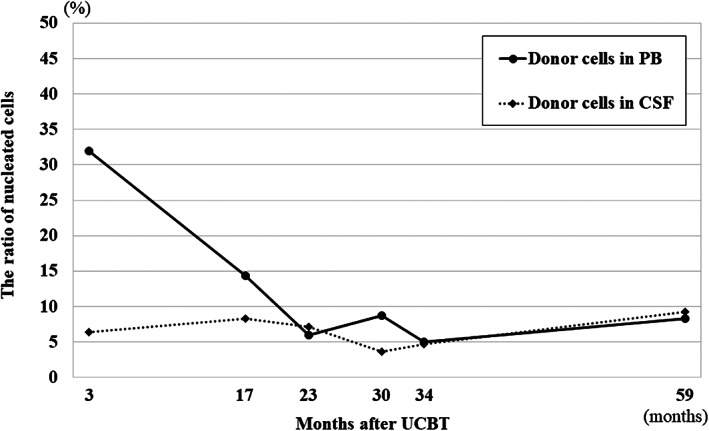
Frequencies of donor cells in peripheral blood (PB) and cerebrospinal fluid (CSF) after unrelated cord blood transplantation (UCBT). Both frequencies remained below 10% over 7 years

The patient was infused with unrelated cord blood containing 7.2 × 10^7^ nucleated cells/kg (1.4 × 10^5^ CD34^+^ cells/kg). Neutrophil engraftment was achieved 16 days after UCBT. Mild acute GVHD (skin, stage 1, grade I) was observed. Chronic GVHD did not develop. There were no infections or virus reactivation of latent infections. Chimerism analysis by fluorescence in situ hybridization (FISH) in the bone marrow showed donor‐dominant mixed chimerism (90.8% of nucleated cells) 20 days after UCBT; however, the proportion of donor cells in the bone marrow decreased to 15.1% of nucleated cells 2 months after UCBT. Thereafter, cyclosporine was discontinued. The low donor chimerism persisted (donor cells in PB, 31.9% of nucleated cells) (Figure 2), while CSF chimerism analysis by FISH detected donor cells in the CSF (6.4% of nucleated cells).

On brain MRI 2 months after UCBT, high‐intensity areas were noted in the right internal capsule, cerebral peduncle, and corpus callosum, in addition to the subcortical white matter of the right frontal and temporal lobes. Mild atrophy of the right frontal and temporal areas was also observed. The Loes score had increased to 8.0 points (Figure 1C).

At 8 months after UCBT, a slight decrease in right periventricular white matter was seen with the involvement of the anterior limb of the internal capsule and the genu of the corpus callosum. The Loes score has decreased slightly to 7.5 points (Figure 1D), and no contrast effect was detected in either MRI. Notably, there were no clinical signs of disease progression. Considering the fact that UCBT stopped the disease progression and the risk of a second HSCT, the parents did not want their child to undergo a second HSCT despite the possibility of future graft failure. Accordingly, the patient was carefully followed up without a second HSCT.

**FIGURE 3:**
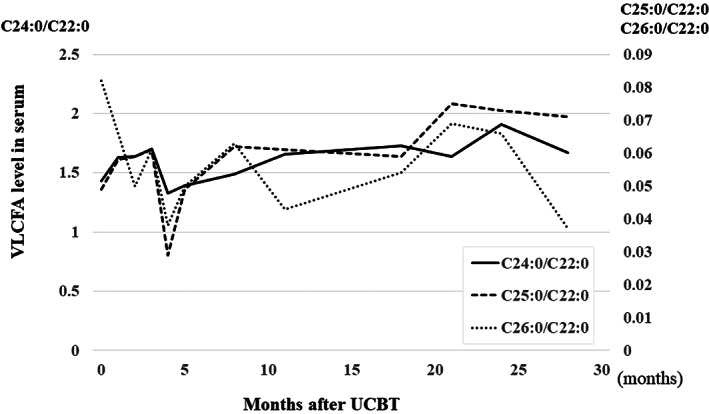
Plasma levels of very‐long‐chain fatty acids (VLCFAs) remained high over 2 years after unrelated cord blood transplantation (UCBT)

The proportion of donor cells remained as low as less than 10% in PB, and was persistently below 10% in the CSF, over 7 years after UCBT (Figure 2). The elevated plasma levels of VLCFAs remained relatively unchanged after HSCT (Figure 3). According to the Wechsler Preschool and Primary Scale of Intelligence III, his full intelligence quotient was 89 at 5 years and 2 months (3 years and 5 months after UCBT). He was diagnosed with attention‐deficit hyperactivity disorder at 7 years of age. However, at 8 years of age, the patient showed no functional disturbance of any extremity, and brain MRI showed no deterioration of white matter lesions at 3 years (Figure 1E) and 7 years (Figure 1F) after UCBT. The patient also had residual adrenal insufficiency and was under corticoid replacement therapy.

## 3 DISCUSSION

We report the case of a patient with CCALD who underwent UCBT at 1 year of age before the appearance of neurological symptoms. Although the ratio of donor‐to‐recipient cells in the CSF and PB remained under 10%, the patient's neurological symptoms or MRI findings did not deteriorate.

The presence of donor cells in the CSF after HSCT for ALD has not been reported. Studies on chimerism analysis of CSF following HSCT for patients with hematological malignancies have revealed the presence of donor cells in the CSF.^17^, ^18^ In patients with an inherited metabolic disorder, such as metachromatic leukodystrophy and globoid‐cell leukodystrophy, HSCT works through a mechanism that depends on donor cells crossing the blood–brain barrier (BBB).^19^

ALD reportedly causes BBB dysfunction, as the deficiency of *ABCD1* activity results in greater adhesion and transmigration of monocytes across the endothelium.^20^ Additionally, Schoenberger et al. reported that sequencing of the *ABCD1* gene in postmortem tissues of CCALD patients who received HSCT revealed the presence of mixed chimerism in some tissues, including brain tissue.^19^ Consistent with these reports, our results suggest that donor cells passed through the BBB and infiltrated the CNS following HSCT.

Longitudinal chimerism studies have shown relatively low donor chimerism in PB. Even with a low chimeric state, our case showed no deterioration of neurological symptoms or MRI findings for 6 years. In a previously reported successful case of hematopoietic stem cell gene therapy, polyclonal reconstitution (with 9%–14% of granulocytes, monocytes, and T and B lymphocytes expressing the ALD protein) was detected in PB.^21^ This result suggests that a small number of cells expressing the ALD protein could suppress demyelination for a certain period of time. In the current case, a small number of donor cells were detected in the CSF for an extended period, and they may be involved in the mechanism that suppresses demyelination in ALD. Contrarily, it has been proposed that HSCT benefits cerebral ALD by replacing or resetting the immune system, thereby halting the neurological inflammatory response.^3^, ^22^ Considering the donor‐dominant mixed chimerism in the bone marrow that was observed early after UCBT, this hypothesis may also explain the present case in which the inflammatory white matter destruction had not progressed, despite the relatively low donor cell count in PB seen later.

A second HSCT is associated with a high risk of transplant‐mediated mortality, and thus determining whether it should be performed is crucial but difficult. VLCFA levels appear to be inadequate as an indicator of the need for a second HSCT because VLCFA levels are higher than the normal values even in successful cases of transplantation.^4^, ^8^ Three out of the four cases of gene therapy mentioned above showed major cognitive decline despite maintaining 5%–10% expression of the transgene in PB, at around 9, 28, and 60 months after the gene therapy, respectively.^23^ The chimerism ratio in PB might not correlate with that in the CSF. In addition, the chimerism ratio in the CSF might not correlate with that in the brain parenchyma. In this case, careful monitoring for clinical manifestations and brain MRI changes over a long period was necessary to avoid missing the opportunity for a second HSCT. Further research is needed to determine whether chimerism analysis in CSF, including cell‐fractionation analysis, can be an indicator in deciding whether to perform a second HSCT.

Recently it was reported that approximately 12% of patients with cerebral ALD experience spontaneous arrest of disease.^24^ In this report, patients who never developed a contrast‐enhancing lesion on MRI scans did not develop a progressive cerebral ALD phenotype. Since no contrast lesions were observed on MRI before UCBT in our case, a halting of the disease process cannot be completely ruled out. However, halting of the disease has been found to be accompanied by MRI abnormalities at a relatively old age (median, 23.3 years; minimum age, 8 years); therefore, it is unlikely in our case with abnormal MRI findings at the young age of 1 year. In addition, his elder brother had CALD; however, CALD cases do not necessarily follow the same course, as the discordant phenotypes are common among siblings with ALD.^25^

In conclusion, this case demonstrates the potential effectiveness of UCBT for ALD, even with low donor chimerism in PB and CSF. However, we should continue to carefully follow up the neurological and MRI findings to avoid missing the opportunity for a second HSCT.

## CONFLICT OF INTEREST

All authors declare that they have no conflict of interest.

## AUTHOR CONTRIBUTIONS

Takahiro Ikeda and Yuta Kawahara assessed the patient and wrote the manuscript. Akihiko Miyauchi performed the neurological assessments. Hitomi Niijima performed the unrelated cord blood transplantation. Rieko Furukawa evaluated the relevant medical imaging and edited the manuscript. Nobuyuki Shimozawa performed the metabolic evaluation and genetic analysis and edited the manuscript. Akira Morimoto, Hitoshi Osaka, and Takanori Yamagata helped assess the patient and edited the manuscript.

## INFORMED CONSENT

All procedures followed were in accordance with the ethical standards of the responsible committee on human experimentation (institutional and national) and with the Helsinki Declaration of 1975, as revised in 2000 (5). Informed consent was obtained from the parents of the patient.

## ETHICS STATEMENT

Not applicable.

## PATIENT CONSENT

Written informed consent was obtained from both parents of the patient.

## Data Availability

The data that support the findings of this study are available from the corresponding author, T.I., upon reasonable request.
